# The Homeobox Protein CEH-23 Mediates Prolonged Longevity in Response to Impaired Mitochondrial Electron Transport Chain in *C. elegans*

**DOI:** 10.1371/journal.pbio.1001084

**Published:** 2011-06-21

**Authors:** Ludivine Walter, Aiswarya Baruah, Hsin-Wen Chang, Heather Mae Pace, Siu Sylvia Lee

**Affiliations:** Department of Molecular Biology and Genetics, Cornell University, Ithaca, New York, United States of America; The Salk Institute for Biological Studies, United States of America

## Abstract

Recent findings indicate that perturbations of the mitochondrial electron transport chain (METC) can cause extended longevity in evolutionarily diverse organisms. To uncover the molecular basis of how altered METC increases lifespan in *C. elegans*, we performed an RNAi screen and revealed that three predicted transcription factors are specifically required for the extended longevity of mitochondrial mutants. In particular, we demonstrated that the nuclear homeobox protein CEH-23 uniquely mediates the longevity but not the slow development, reduced brood size, or resistance to oxidative stress associated with mitochondrial mutations. Furthermore, we showed that *ceh-23* expression levels are responsive to altered METC, and enforced overexpression of *ceh-23* is sufficient to extend lifespan in wild-type background. Our data point to mitochondria-to-nucleus communications to be key for longevity determination and highlight CEH-23 as a novel longevity factor capable of responding to mitochondrial perturbations. These findings provide a new paradigm for how mitochondria impact aging and age-dependent diseases.

## Author Summary

Mitochondria have long been associated with aging and age-related diseases. Recent research has shown that a slight dampening of mitochondrial function can dramatically increase the lifespan of a wide range of organisms, suggesting that a similar mechanism likely operates in humans. The molecular basis of this observation is largely unknown, however. Uncovering the genes that allow altered mitochondrial function to impact longevity will give us important new insights into how mitochondria affect the aging process and will pave the way for future therapeutic developments aiming to improve healthy aging and to treat age-related diseases. Here, we used an RNAi screen in the genetic model organism *C. elegans*, a nematode worm, to uncover how altered mitochondrial function can modulate longevity. We found that in order for mitochondria to affect lifespan, they must communicate with several unique transcription factors in the nucleus. Notably, we discovered that the putative homeobox transcription factor CEH-23, which has not previously been implicated in longevity determination, is able to respond to changes in mitochondrial function and in turn causes an extension in lifespan.

## Introduction

Alterations of mitochondrial function broadly impact animal physiology and physiopathology, including aging and age-related diseases. Correlative evidence has long demonstrated that mitochondrial function gradually declines with age, while oxidative damage and mitochondrial DNA mutations accumulate [1]–[4]. Interestingly, recent studies revealed that reduced mitochondrial electron transport chain (METC) function can cause substantial longevity increase in a wide range of organisms. In yeast, some respiration-deficient strains exhibit lifespan increase [5]. In worms, particular METC mutations can greatly extend lifespan. These include mutations in *isp-1*, which encodes the iron sulfur protein of Complex III [6], and in *clk-1*, which encodes the hydroxylase protein necessary for the biosynthesis of the METC electron transporter coenzyme Q [7]. Furthermore, RNAi knockdown of several sub-units of the METC also results in greater longevity in worms [8]–[13] and in fruit flies [14],[15]. In mice, heterozygous loss of the mouse *clk-1* homolog (*mclk-1*) [16] as well as defects in the assembly of the complex IV of the METC [17] extend lifespan. Therefore, the observation that reduced mitochondrial function can prolong lifespan appears highly conserved among evolutionarily diverse species and is likely to be relevant to human physiology.

Not surprisingly, METC mutations can also lead to deleterious manifestations such as developmental arrest or shorter lifespan. For instance, *mev-1* mutant worms, which harbor a mutation in the subunit C of the Complex II, are characterized by a drastic lifespan shortening compared to wild-type worms [18]. Additionally, genetic manipulations that reduce mitochondrial function are often associated with slower physiological rates, regardless of whether they cause a prolonged or shortened longevity phenotype [5]–[7],[12],[14],[15],[19]. In many instances, the severity of the mitochondrial perturbations correlates with their effects on lifespan [12],[20]. A model has emerged in which moderate mitochondrial impairment positively impacts longevity until a threshold is reached, beyond which animal survival is compromised.

In yeast, impaired mitochondria can signal the nucleus through a retrograde signaling pathway that leads to nuclear gene expression changes that in turn extend longevity [21]–[23]. Similarly, in mammalian cells, changes in mitochondrial state trigger a retrograde signaling pathway that results in nuclear transcription factors activation and metabolic or stress-related responses ([24],[25] and [26] for review), but its effect on lifespan is unknown. In general, the molecular mechanisms that enable altered METC to positively impact longevity in multicellular organisms are still unclear. Emerging evidence indicates that longevity extension induced by METC impairment does not correlate with a decrease in oxidative stress response [1],[3],[27] or respiratory capacities [6],[14],[19],[28]. Therefore, the mitochondrion is likely to play a causative role in longevity determination via novel mechanisms that are yet to be uncovered. We hypothesized that, similarly to what was shown in yeast, altered METC in *C. elegans* results in signaling that impinges upon specific transcription factor(s) to promote longevity. Our study revealed the identity of several nuclear transcription factors that are specifically important for the longevity increase associated with altered METC in *C. elegans*, and points to a novel molecular basis by which mitochondria impact longevity in animals.

## Results

### Targeted RNAi Screen for Putative Transcription Factors That Suppress the Longevity Phenotype of the Long-Lived *isp-1;ctb-1* Mitochondrial Mutant

**Figure 1:**
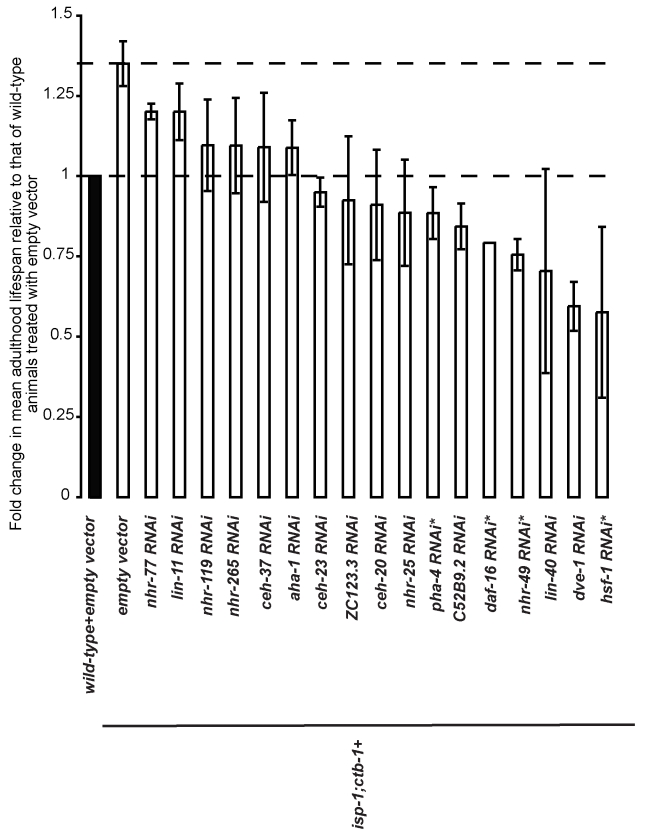
A targeted RNAi screen identifies 17 RNAi candidates that decrease the lifespan of the *isp-1;ctb-1* mutant. The mean fold change in lifespan relative to that of wild-type worms treated with empty vector (black bar, value set to 1) was calculated for *isp-1;ctb-1* mutant treated with empty vector or with each RNAi clone tested. The mean fold changes (± s.d.) presented are averaged from at least three independent experiments. Only the RNAi clones that decrease the lifespan of the *isp-1;ctb-1* mutant by at least 10% are represented (see Table 1). Among them, 13 represent transcription factors not previously known to function in aging and four (indicated with asterisks) are transcription factors already known to affect lifespan in *C. elegans*. Quantitative data and statistical analyses for the experiments shown here are included in Tables 1 and S1.

To identify the putative transcription factor(s) that mediate the lifespan increase of worms with reduced METC function, we employed a targeted RNAi screening approach using the Transcription Factor RNAi Library (Gene Service Inc.), which covers ∼41% of the predicted transcription factors in *C. elegans* [29]. We performed the RNAi screen using the *isp-1;ctb-1* mitochondrial mutant, which exhibits a robust lifespan increase with only mild delays in rates of developmental processes [6]. We systematically inactivated, by RNAi feeding [30] from the time of hatching, each of the 387 transcription factors in the library and looked for RNAi targets that suppress the prolonged lifespan of the *isp-1;ctb-1* mutant worms. From the primary screen, we identified 32 RNAi candidates that caused a decrease in the *isp-1;ctb-1* mutant lifespan by at least 15% when compared to empty vector control RNAi (*p*≤0.001, log-rank test). The 32 primary candidates were then retested in at least two independent trials (Figure S1). To select the RNAi candidates that consistently suppressed the prolonged lifespan of *isp-1;ctb-1*, we used two different methods of data analysis. The percentage of lifespan suppression caused by each RNAi clone was either calculated after pooling the data from the independent trials and comparing the mean lifespan by stratified log-rank test (*p*≤0.001) or averaged after a comparison of mean lifespan within each trial by log-rank-test (*p*≤0.001) (see Experimental Procedures). The results revealed that 17 RNAi candidates consistently shortened the lifespan of the *isp-1;ctb-1* mutant by more than 10% (Tables 1 and S1). Among these RNAi candidates, four correspond to transcription factors previously shown to be important for longevity maintenance in *C. elegans*: the heat shock factor HSF-1 [31], the nuclear hormone receptor NHR-49 [32], and the forkhead factors DAF-16 [33] and PHA-4 [34]. The other 13 candidate transcription factors have not been previously implicated in directly affecting *C. elegans* longevity. Many transcription factor families are represented within these 13 candidates, with an overrepresentation of the homeobox class (∼40% of the candidate clones are homeobox proteins, whereas ∼10% of all the clones tested in the RNAi screen are predicted to be homeobox proteins). Under the conditions of our lifespan assays, *isp-1;ctb-1* mutants typically exhibit a 1.35-fold increase in mean lifespan compared to wild-type (Figure 1). Thus, RNAi clones that decrease the lifespan of *isp-1;ctb-1* mutant worms by ∼30% (*nhr-119*, *nhr-265*, *ceh-37*, *aha-1*, *ceh-*23, *ZC123.3*, *ceh-20*, and *nhr-25*; Figure 1) are of special interest since they represent RNAi knockdowns that restore the lifespan of the *isp-1;ctb-1* mutant to that of wild-type worms. Although *cep-1*, the *C. elegans* homolog of the tumor suppressor *p53*, has previously been shown to be required for the extended lifespan of *isp-1* mutant [35], *cep-1* RNAi did not exhibit significant lifespan suppression in our screen. This might be due to *cep-1* RNAi only partially knocked down *cep-1* in our screen conditions or that *cep-1* is not required for longevity increase of *isp-1;ctb-1* mutant.

**Table 1:** A targeted RNAi screen identifies 17 RNAi candidates that decrease the prolonged lifespan of the METC mutant *isp-1;ctb-1* by at least 10%.

Gene Name	Transcription Factor Family	Number of Independent Lifespan Assays	Stratified Log-Rank Test (*p*≤0.001) for Pooled Independent Lifespan Assay	Log-Rank Test (*p*≤0.001) for Each Independent Lifespan Assay
			Decrease of *isp-1*; *ctb-1* Mutant Mean Lifespan (%)	Average Decrease of *isp-1*; *ctb-1* Mutant Mean Lifespan (Mean % ± S.D.)
*dve-1*	Homeobox	3	−55	−56±2
hsf-1*	Heat shock	2		−46±17
*lin-40*	Histone deacetylase	3	−48	−46±25
nhr-49*	Nuclear hormone receptor	2	−46	−45±1
daf-16*	Forkhead	1		−44
*C52B9.2*	Ets domain	4	−38	−36±8
*ZC123.3*	Homeobox	3	−37	−34±14
*ceh-20*	Homeobox	2	−32	−33±12
*nhr-25*	Nuclear hormone receptor	5	−32	−28±21
*ceh-23*	Homeobox	3	−29	−26±8
pha-4*	Forkhead	2		−20±17
*nhr-119*	Nuclear hormone receptor	2	−15	−13±4
*nhr-265*	Nuclear hormone receptor	2	−12	−13±3
*ceh-37*	Homeobox	4	−12	−14±3
*lin-11*	Homeobox	5	−11	−15±7
*aha-1*	Aryl-hydrocarbon receptor nuclear translocator	5	−11	−16±8
*nhr-77*	Nuclear hormone receptor	2	−11	−12±6

The RNAi candidates identified in the primary screen were retested in the number of independent lifespan assays indicated. The lifespan data from the separate trials were either pooled, compared by a stratified log-rank test (*p*≤0.001), and the percentage of mean lifespan decrease calculated or compared by log-rank test (*p*≤0.001) within each trial and the percentage of mean lifespan decrease was averaged. Only the RNAi clones that decrease the lifespan of the *isp-1;ctb-1* mutant by at least 10% in the two methods of analysis are represented. Among them, 13 represent transcription factors not previously known to function in aging and four (indicated with asterisks) are transcription factors already known to affect lifespan in *C. elegans*: HSF-1 [31], NHR-49 [32], DAF-16 [33], and PHA-4 [34]. Quantitative data and statistical analyses for the experiments shown here are included in Table S1.

### Identification of Transcription Factors That Specifically Mediate the Effects of METC Mutations on Longevity

We reasoned that if the METC affects lifespan through specific transcription factors, RNAi knockdown of those transcription factors should decrease the *isp-1;ctb-1* mutant lifespan to an extent greater than that of wild-type worms or other longevity mutants thought to act independently of mitochondria. To assay the specificity of the transcription factors identified in our primary screen (Figure 1 and Table 1), we tested the 13 RNAi candidates for an effect on the lifespan of wild-type worms, the short-lived FOXO transcription factor *daf-16* mutant [33],[36], and the long-lived phosphatidyl inositol 3-kinase *age-1* mutant [37],[38] that exhibits a lifespan increase as robust as that of the *isp-1;ctb-1* mutant. Both *daf-16* and *age-1* represent components of the insulin/insulin-like growth factor signaling (IIS) pathway, which is thought to act mostly independently of the METC to affect lifespan in *C. elegans* [39]. As expected, several of the RNAi candidates (*dve-1*; *lin-40*; *nhr-49*; *ceh-20*; *lin-11*; and *nhr-77*) appeared to non-discriminately shorten lifespan in all strains tested (Tables 2 and S1), suggesting that the corresponding transcription factors are broadly required for survival. Interestingly, four RNAi clones (*ZC123.3*; *nhr-119*; *ceh-37*; and *aha-1*) affected wild-type and *isp-1;ctb-1* mutant worms' lifespan to the same extent but exerted only a moderate or no effect on *daf-16* and *age-1* mutant longevity (Tables 2 and S1). Those transcription factors are unlikely to specifically mediate the effects of METC mutations on longevity as their knock-down affected longevity of wild-type and *isp-1;ctb-1* worms to a similar extent. Interestingly, four other RNAi clones, targeting the predicted transcription factors C52B9.2, NHR-25, CEH-23, and NHR-265, substantially shortened the lifespan of the *isp-1;ctb-1* mutant, but either had a lesser or no effect on the other strains tested including wild-type worms (Tables 2 and S1), suggesting that the corresponding transcription factors are preferentially required for the longevity of *isp-1;ctb-1* mutants. To test whether these four transcription factors (C52B9.2, NHR-25, CEH-23, and NHR-265) may contribute to the longevity effect caused by different METC perturbations, we next tested the effects of their RNAi knockdown on the lifespan of two additional long-lived mitochondrial mutant worms, *isp-1* and *clk-1*, which display a similar degree of lifespan extension (Figure S1). Among the four candidates tested, *nhr-265* is the only one that is specifically required for *isp-1;ctb-1* mutant worms to exhibit greater lifespan but not for the other long-lived mitochondrial mutants tested (Tables 2 and S1). The other three factors (C52B9.2, *ceh-23*, and *nhr-25*) appear to be important for the longevity effects of all three long-lived METC mutant strains (Tables 2 and S1).

**Table 2:** Genetic interaction analyses of the RNAi candidates in several longevity mutant strains.

	Variation of Mean Adulthood Lifespan (%)							
Gene	*isp-1;ctb-1*	*Wild-type*	*daf-16*	*age-1*	*isp-1*	*clk-1*	*mev-1*	*eat-2*
*dve-1*	−55	−53	−35	−53				
*lin-40*	−48	−18	−18	−57				
*nhr-49*	−46	−56	−38	−32				
*ceh-20*	−32	−12	−10	−23				
*lin-11*	−11	−9	−4	−18				
*nhr-77*	−11	−7	−10	−15				
ZC123.3	−37	−45	−12	*n.s.*				
*nhr-119*	−15	−10	*n.s.*	*n.s.*				
*ceh-37*	−12	−8	*n.s.*	*n.s.*				
*aha-1*	−11	−10	−5	*n.s.*				
C52B9.2	−38	−23	−12	*n.s.*	−30	−35	−12	−30
*nhr-25*	−32	−8	*n.s.*	+14	−46	−29	*n.s.*	−44
*ceh-23*	−29	+12	+23	*n.s*	−29	−32	*n.s.*	*n.s.*
*nhr-265*	−12	+4	*n.s.*	*n.s.*	*n.s.*	*n.s.*	*n.s.*	*n.s.*

The effects of the RNAi candidates identified by our screen on the lifespan of different *C. elegans* mutant strains are shown as a percentage variation of the mean adult lifespan when compared to empty vector RNAi (stratified log-rank, *p*≤0.001). The mean adult lifespan for each RNAi candidate and for empty vector RNAi was obtained by pooling at least two independent lifespan assays. RNAi clones of *C52B9.2*, *nhr-25*, and *ceh-23* specifically shortened the lifespan of all METC mutants tested (*isp-1;ctb-1*, *isp-1*, and *clk-1* mutants). Among them, *ceh-23* RNAi is the only one that does not affect the lifespan of *eat-2* mutants. Quantitative data and statistical analyses for the experiments shown here are included in Table S1. − indicates a percentage decrease and + indicates a percentage increase of the mean adulthood lifespan when the mean lifespan of worms fed with the RNAi candidate and those fed with the empty vector RNAi were statistically significantly different (stratified log-rank, *p*≤0.001). *n.s.* indicates that the mean lifespan of worms fed with the RNAi candidate and those fed with the empty vector RNAi were not statistically significantly different (stratified log-rank, *p*≥0.001). Underlined gene names indicate genes that, when down-regulated, have a stronger effect on *isp-1; ctb-1* mutant worms' lifespan than on wild-type worms' lifespan.

We also tested whether RNAi of these four factors affected the lifespan of the short-lived *mev-1* mitochondrial mutant worms. Three of the RNAi clones (*nhr-25*, *ceh-23*, and *nhr-265*) did not affect the lifespan of *mev-1* mutants. C52B9.2 RNAi shortened *mev-1* mutant lifespan (−12%) but to a lesser degree than that of wild-type worms (−23%) (Tables 2 and S1). All together, our data suggest that specific transcription factors play an important role in the longevity of long-lived mitochondrial mutants without affecting the lifespan phenotype of short-lived mitochondrial mutants.

Next, as the mitochondrial *clk-1* mutation and the genetic mimic of dietary restriction *eat-2* mutation have previously been shown to act in a similar genetic pathway [40], we examined the three transcription factors (C52B9.2, *ceh-23*, and *nhr-25*) that are important for the longevity of the *clk-1* mutant (Tables 2 and S1) for an effect on the lifespan of the *eat-2* mutant worms. RNAi knockdown of C52B9.2 and *nhr-25* decreased the lifespan of the *eat-2* mutant to a greater extent than that of wild-type worms (respectively −30% and −44% in *eat-2* mutant versus −23% and −8% in wild-type; *p*≤0.001, log-rank test; Table 2). In contrast, knockdown of *ceh-23* had no significant consequence on the lifespan of *eat-2* mutant worms (Table 2). Therefore, all together, our targeted RNAi screen data identified the homeobox protein CEH-23 as a candidate uniquely required for the extended longevity of mitochondrial mutants.

### The Homeobox Transcription Factor CEH-23 Mediates the Longevity Effects of METC Mutations

**Figure 2:**
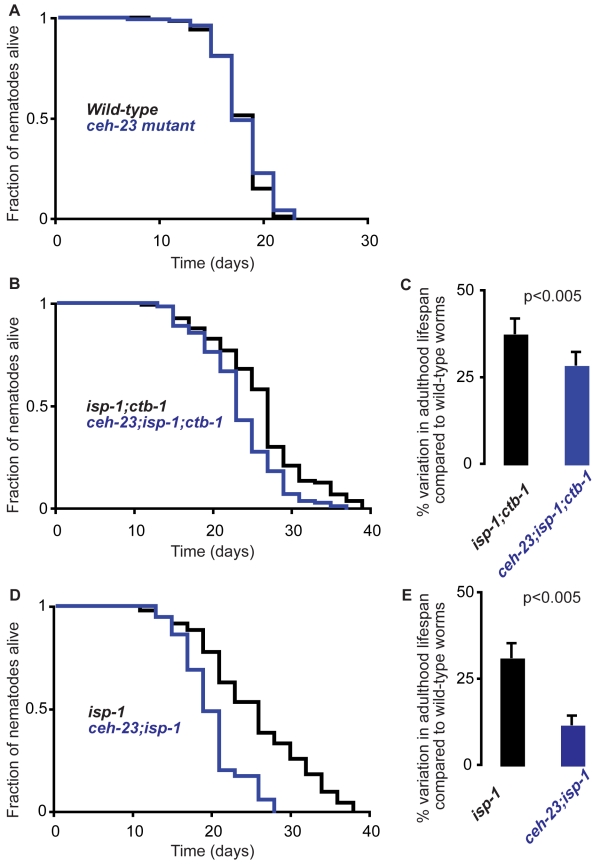
*ceh-23* mediates the extended longevity phenotype of mitochondrial mutant worms. (A) Wild-type worms (black curve) exhibited the same lifespan as *ceh-23(ms23)* mutants (blue curve). (B) *isp-1(qm150);ctb-1(qm189)* mutants (black curve) exhibited longer mean and maximum lifespan than *ceh-23(ms23);isp-1(qm150);ctb-1(qm189)* mutants (blue curve). (C) *isp-1(qm150);ctb-1(qm189)* mutants exhibited a greater lifespan extension (37.8±4.1%) than *ceh-23(ms23);isp-1(qm150);ctb-1(qm189)* mutants (28.6±3.7%) when compared to wild-type worms; *p*≤0.005, mixed model analysis with *ceh-23* mutation as fixed effect and experiment as random effect. (D) *isp-1(qm150)* mutants (black curve) exhibited longer mean and maximum lifespan than *ceh-23(ms23);isp-1(qm150)* mutants (blue curve). (E) *isp-1(qm150)* mutants exhibited a greater lifespan extension (31.3±4.0%) than *ceh-23(ms23);isp-1(qm150)* mutants (11.8±2.4%) when compared to wild-type worms; *p*≤0.005, mixed model analysis with *ceh-23* mutation as fixed effect and experiment as random effect. Data from one representative experiment are shown in Panels A, B, and D. Data from at least four pooled experiments are shown in Panels C and E. Quantitative data and statistical analyses for all the experiments performed are included in Table S2.

Although based on sequence alignment (BLAST), the *ceh-23* RNAi clone used in the screen is specific, we constructed three additional RNAi constructs targeting different regions of the *ceh-23* gene to rule out possible off-target effects of the *ceh-23* RNAi from the Gene Service Library (Figure S2). All three of the newly generated RNAi constructs significantly (*p*≤0.001, log-rank test) suppressed the longevity of *clk-1*, *isp-1;ctb-1*, and *isp-1* mutant worms to a similar extent and had no effect on the lifespan of wild-type worms, *age-1*, *daf-16*, and *eat-2* mutant worms (Figure S3 and unpublished data). Taken together, our data indicate that knockdown of *ceh-23* specifically shortens the lifespan extension of the long-lived mitochondrial mutant worms and is not likely to compromise the general health of the worm. To avoid any pitfalls associated with the RNAi strategy [41], we next examined the *ceh-23(ms23)* mutant, which harbors a deletion that covers 75% of the *ceh-23* gene, including half of the homeobox domain ([42] and Figure S2). While the *ceh-23* single mutant exhibited wild-type lifespan [43], we found that loss of *ceh-23* decreased the lifespan of *isp-1;ctb-1* mutant by ∼10% and that of *isp-1* by ∼20% (Figure 2 and Table S2). The *ceh-23* genetic mutation suppressed the METC mutants' lifespan to a similar degree as the three *ceh-23* RNAi constructs we generated in the lab (Figure S2), but to a lesser degree compared to *ceh-23* RNAi construct from the screen library. We purposely performed the screen with a mixture of RNAi colonies for each RNAi construct; thus, it is possible that the *ceh-23* RNAi bacteria used in the screen had unexpected off-target effects that caused greater shortening of METC mutant lifespan. Overall, the *ceh-23* mutant data corroborated the *ceh-23* RNAi results and indicated that *ceh-23* inactivation specifically shortens the lifespan extension of the long-lived mitochondrial mutants without compromising the general health of the worm. Taken together, our data highlight a crucial role of CEH-23 in mediating the extended longevity caused by METC mutations.

### CEH-23 Does Not Mediate the Development and Fertility Defects or the Paraquat Resistance Induced by *isp-1* Mutation

Having established that CEH-23 is required for the prolonged lifespan phenotype of mitochondrial mutants (Figure 2), we next asked whether CEH-23 also plays a role in the other phenotypes often associated with reduced mitochondrial function, such as slower development rate and reduced self-brood size [6]. We examined the effects of *ceh-23* deletion and *ceh-23* RNAi inactivation on the development rate and brood size of mitochondrial mutants and wild-type worms. We found that *ceh-23* mutants and *ceh-23* RNAi treated worms exhibit development rate and brood size indistinguishable from that of wild-type worms (Table 3). As previously published [6], *isp-1* and *isp-1;ctb-1* mutant worms develop substantially slower and produce a much lower brood compared to wild-type worms (Table 3). Importantly, *ceh-23;isp-1* and *ceh-23;isp-1;ctb-1* mutants have development rate and brood size similar to those of *isp-1* or *isp-1;ctb-1* mutants, respectively. Similar results were obtained when *ceh-23* was knocked down by RNAi in the *isp-1;ctb-1* mutant (Table 3). These data indicate that CEH-23 is unlikely to participate in the regulation of development rate and brood size in mitochondrial mutants.

**Table 3:** Effects of knocking down *ceh-23* on the rate of development, self-brood size, and resistance to paraquat.

	Development and Brood Size	
	Number of Hours to Reach Adulthood	Self-Brood Size
	Mean ± SD (Hours)	Mean ± SD (Number of Worms)
Strain	(Sample size)	(Sample size)
wild-type	56±6	350±65
	(533)	(10)
*ceh-23(ms23)*	57±6	396±37
	(527)	(10)
*isp-1(qm150);ctb-1(qm189)*	76±4*	203±67*
	(388)	(10)
*ceh-23(ms23);isp-1(qm150);ctb-1(qm189)*	77±6*	216±52*
	(403)	(10)
*isp-1(qm150)*	109±6*	124±13*
	(201)	(10)
*ceh-23(ms23);isp-1(qm150);*	106±6*	128±25*
	(194)	(9)
wild-type+empty vector	53±7	358±45
	(343)	(5)
wild-type+*ceh-23 RNAi*	53±6	289±47
	(336)	(5)
*isp-1(qm150);ctb-1(qm189)+empty vector*	82±5*	217±22*
	(192)	(5)
*isp-1(qm150);ctb-1(qm189)+ceh-23 RNAi*	95±8*	186±27*
	(211)	(5)

	Survival on Paraquat	
	Survival	
	Mean ± SD (Days)	
Strain	(Sample Size)	Variation in Survival When Compared to Wild-Type Worms (%)
wild-type	2.47±0.08	
	(82)	
*ceh-23(ms23)*	2.57±0.06	4
	(85)	
*isp-1(qm150)*	3.85±0.09‡	56
	(109)	
*ceh-23(ms23);isp-1(qm150)*	4.13±0.12‡	67
	(101)	

Data shown are the average of two independent experiments.

*Significantly different when compared to wild-type worms, *p*≤0.05 Student's *t* test.

**‡:** Significantly different when compared to wild-type worms, *p*≤0.001 log-rank test.

Whereas increased longevity in *C. elegans* is often associated with stress resistance [44], an interesting characteristic of the long-lived mitochondrial mutant worms is that they do not exhibit consistent resistance to different stresses, especially oxidative stress [3],[8]. In examining different oxidative stress conditions, we noticed that *isp-1* mutant worms exhibited different responses to the superoxide-inducing agent paraquat depending on the developmental stage at which they were exposed to the chemical (Figure S4). We found that *isp-1* mutant worms exhibited a better mean survival than wild-type worms when paraquat treatment was initiated at the L4 stage (Figure S4 and Table 3). Under this condition, loss of *ceh-23* had no effect on the mean survival of wild-type or *isp-1* mutants worms (Table 3), indicating that CEH-23 is not required for the paraquat resistance of *isp-1* mutant worms under this assaying condition.

Taken together, the results suggest that CEH-23 is specifically important for the prolonged longevity, but not the slow rates of development and reproduction, or the oxidative stress resistance associated with the *isp-1* mutation.

### The Expression of the Homeobox Transcription Factor CEH-23 Is Enriched in the Nuclei of Intestinal and Neuronal Cells

**Figure 3:**
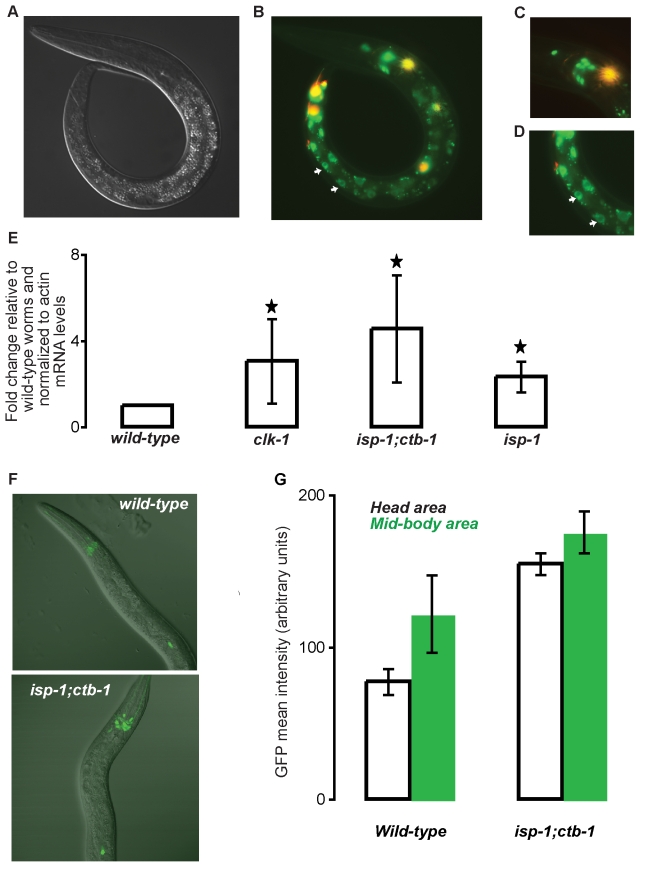
*ceh-23* expression is enriched in the nuclei of intestinal and neuronal cells and is increased in response to altered mitochondrial function. (A) DIC image of a *rwEx19[gfp::ceh-23+mec-7::rfp]* transgenic worm. (B) Overlay of a GFP-channel image (shown in green) and a RFP-channel image (shown in red) of a *rwEx19[gfp::ceh-23+mec-7::rfp]* transgenic worm. *mec-7::rfp* transgene was used as a co-injection marker. The green fluorescence likely represents the authentic expression pattern of CEH-23. The white arrows point to the nuclei of intestinal cells. The image shown is a representative of data obtained with two independent lines. (C) Enlarged overlay (GFP and RFP) image of the head of a *rwEx19[gfp::ceh-23+mec-7::rfp]* transgenic worm showing some of the neurons expressing CEH-23. (D) Enlarged overlay (GFP and RFP) image of the posterior intestine of a *rwEx19[gfp::ceh-23+mec-7::rfp]* transgenic worm showing enriched GFP expression in the nuclei of some of the intestinal cells (white arrows). (E) mRNA expression level of *ceh-23* in wild-type worms and *clk-1(e2519)*, *isp-1(qm150);ctb-1(qm189)*, and *isp-1(qm150)* mutant worms. The mean *ceh-23* mRNA levels normalized to actin mRNA levels are shown. The mean normalized mRNA levels in wild-type worms were set as 1. Data shown were pooled from three independent experiments, *p*≤0.05, Student *t* test. (F) GFP expression in *gmls-18[ceh-23::gfp]* and in *isp-1;ctb-1;gmls-18[ceh-23::gfp]* transgenic mutant worms at L4 stage was visualized using confocal microscopy. CEH-23::GFP expression is shown in green and fluorescent image was merged with DIC image. (G) Quantification of the GFP expression levels in *gmls-18[ceh-23::gfp]* and in *isp-1;ctb-1;gmls-18[ceh-23::gfp]* transgenic mutant worms. Data shown were obtained from four representative worms.

To further characterize CEH-23, we examined its expression pattern in wild-type and mitochondrial mutant worms. Because an antibody capable of recognizing endogenous CEH-23 is not available, we established multiple independent transgenic lines overexpressing an N-terminal GFP-fused CEH-23. This construct is likely to reflect the authentic CEH-23 expression pattern as it contains the entire coding region of *ceh-23* (including introns), as well as its predicted promoter and 3′-UTR (Figure S2). Interestingly, the expression of CEH-23::GFP was restricted to a handful of neurons and the intestine of the worm (Figure 3A–D represent the data obtained in two independent lines). Although we have not identified the neurons in which CEH-23 is expressed, the observed neuronal expression of our CEH-23::GFP construct (Figure 3B and 3C) resembles that of a previously published GFP construct fused to a partial CEH-23 (*gmIs18[ceh-23::gfp]*; [45],[46] and Figure 3F), which was reported to express in the pair of CAN neurons in the central body and 12 sets of sensory neurons in the head (10 pairs) and the tail (2 pairs). While the neuronal expression of CEH-23 was found in all the transgenic worms, the intestinal expression was only detected in ∼50% of the transgenic worms with a stronger expression in the posterior intestine (Figure 3D). We currently do not understand what determines the mosaicism of the intestinal expression. However, it is of note that intestinal localization of *ceh-23* has been previously observed using a promoter(*ceh-23*)::GFP fusion [47]. Enriched nuclear localization of CEH-23 was detected in both the neurons and the intestine, consistent with a putative role of CEH-23 as a transcription factor. No systematic differences in CEH-23 expression pattern were detected in *isp-1;ctb-1* or *isp-1* mutant worms compared to wild-type animals (unpublished data), suggesting that METC mutations are unlikely to affect the tissues nor the cellular compartments in which CEH-23 is expressed.

### CEH-23 Expression Is Increased in Long-Lived Mitochondrial Mutants

**Figure 4:**
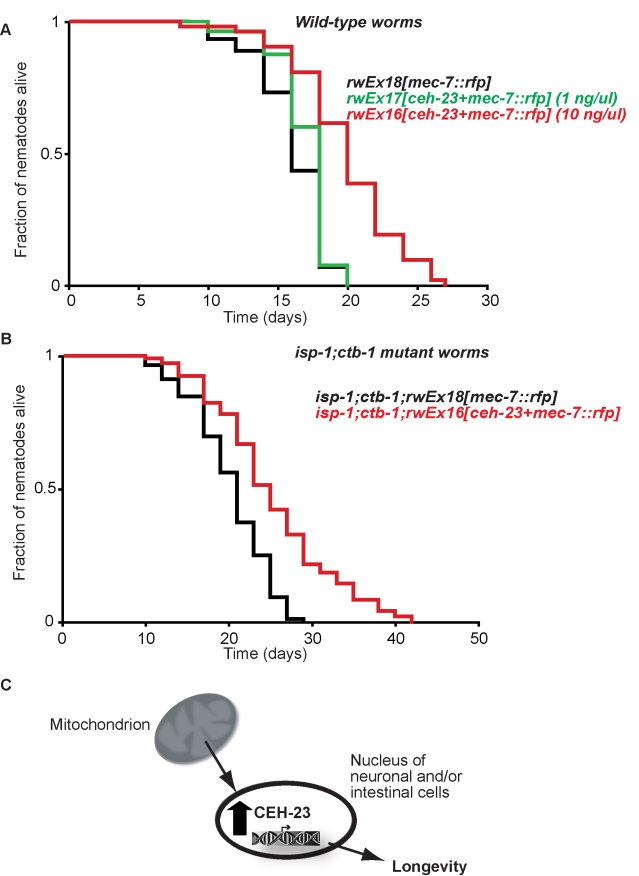
*ceh-23* modulates *C. elegans* lifespan and responds to altered mitochondrial respiratory chain. (A) Worms transformed with 10 ng/µl of *ceh-23* (red curve) *rwEx16[ceh-23+mec-7::rfp]* showed an increased lifespan (*p*≤0.001, log-rank test) when compared to *rwEx18[mec-7::rfp]* control worms (black curve). By contrast, worms transformed with 1 ng/µl of *ceh-23* (green curve) *rwEx17[ceh-23+mec-7::rfp]* showed only marginal lifespan increased (*p* = 0.036, log rank test) when compared to *rwEx18[Pmec-7::rfp]* control worms (black curve). (B) *isp-1(qm150);ctb-1(qm189);rwEx16[ceh-23+mec-7::rfp]* worms (red curve) showed increased lifespan (*p*≤0.001, log-rank test) when compared to *isp-1(qm150);ctb-1(qm189);rwEx18[mec-7::rfp]* worms (black curve). Data are from one representative experiment that was repeated at least three times with similar results. Quantitative data and statistical analyses for all the experiments are included in Table S3. (C) A model of how CEH-23 mediates the longevity effects of altered mitochondrial respiratory chain. We propose that impaired METC induces upregulation of the nuclear transcription factor CEH-23, which results in CEH-23-mediated gene expression changes that affect longevity.

We next compared the expression levels of *ceh-23* in wild-type and in long-lived METC mutants using quantitative reverse-transcription PCR (qRT-PCR). Interestingly, we found that *ceh-23* mRNA levels were elevated in the *isp-1;ctb-1*, *isp-1*, and *clk-1* mutant worms compared to wild-type worms (Figure 3E, *p*≤0.05, Student's *t* test). Our finding is consistent with a recent microarray study indicating that *ceh-23* mRNA level is elevated upon certain mitochondrial perturbations [48]. To further corroborate our qRT-PCR data, we compared *ceh-23::gfp* expression in wild-type and in the *isp-1;ctb-1* mutant. We used the *gmIs18[ceh-23::gfp]* strain (see above) for this imaging experiment due to its stable and homogenous expression among worm populations of the same genotype. Using confocal microscopy, we detected a substantially higher level of *ceh-23::gfp* expression in the *isp-1;ctb-1* mutant compared to wild-type worms throughout different developmental stages of the worms (Figure 3F–G and Figure S5). Our data indicate that METC mutations can lead to increased CEH-23 expression and suggest that CEH-23 is able to respond to altered mitochondrial function (Figure 4C).

### The Nuclear Homeobox Transcription Factor CEH-23 Modulates *C. elegans* Longevity

Since mitochondrial mutant worms require the presence of *ceh-23* to maintain their lifespan (Figure 2) and exhibit higher levels of *ceh-23* (Figure 3), we hypothesized that overexpression of *ceh-23* might confer a long-lived phenotype, possibly by mimicking induced *ceh-23* expression in the mitochondrial mutants. To test this, we established multiple independent transgenic lines overexpressing *ceh-23* (*ceh-23(o/e)*) using a construct identical to the one we used for examining CEH-23 expression above, except that it lacks the GFP fusion (Figure S2). We tested the lifespan of six independent extrachromosomal *ceh-23(o/e)* lines and observed that three of the lines showed a consistent and significant lifespan increase compared to the co-injection marker only transgenic controls (Figure 4A and Table S3, *p*≤0.001 log-rank test). In the three other lines tested, overexpressing CEH-23 significantly increased longevity only in half of the experiments (Table S3). Mosaicism among transgenic lines is commonly observed in *C. elegans*. The differences in longevity of the different transgenic lines assayed may be due to silencing of the transgene in some of the lines upon propagation [49] and/or different levels of CEH-23 expression in the different lines. To further address this point, we generated transgenic worms expressing lower levels of CEH-23 by transforming worms with 10 times less DNA (1 ng/µl instead of 10 ng/µl). Overall, the 1 ng/µl lines exhibited only marginal lifespan increase, indicating that the effect of CEH-23 overexpression on the longevity of otherwise wild-type worms appears to be proportional to the quantity of DNA injected into worms (Figure 4A and Table S3). To further ensure that the lifespan increase phenotype we observed was due to elevated CEH-23 expression, we performed two additional control experiments. First, we treated one *ceh-23(o/e)* line (*rwEx16*, line 5) that consistently showed significant lifespan extension with *ceh-23* RNAi and showed that the lifespan increase phenotype was abrogated when *ceh-23* was knocked down in the transgenic worms (Figure S6A). Second, we introduced one *ceh-23(o/e)* line (*rwIs21*), which showed no obvious lifespan increase in wild-type background, into the *ceh-23;isp-1* mutant background. Overexpression of *ceh-23* in this scenario was able to significantly prolong the normally shortened lifespan of the *ceh-23;isp-1* mutant worms, suggesting that re-expression of functional *ceh-23* was able to revert the lifespan suppression caused by *ceh-23* mutation (Figure S6B).

Because overexpressing *ceh-23* is sufficient to increase wild-type lifespan (Figure 4A), we hypothesized that increased *ceh-23* levels contribute to the longevity increase of mitochondrial mutants and that elevated *ceh-23* expression and METC mutations increase lifespan through common mechanisms. With such a model, one might expect that overexpressing CEH-23 in METC mutants will not cause further extension of lifespan. To test our hypothesis, we examined the effect of CEH-23 overexpression in *isp-1;ctb-1* mutant worms using three independent *isp-1;ctb-1;ceh-23(o/e)* lines. Two *isp-1;ctb-1;ceh-23(o/e)* lines further increased lifespan when compared to the *isp-1;ctb-1;mec-7::rfp* control lines (Figure 4B and Table S3, *isp-1(qm150);ctb-1(qm189);rwEx16[ceh-23+mec-7::rfp]*, lines 4 and 3 versus control *isp-1(qm150);ctb-1(qm189);rwEx18[mec-7::rfp]* line 3, *p*≤0.001, log-rank test). Another *isp-1;ctb-1;ceh-23(o/e)* line either exhibited the same or a shorter lifespan than the *isp-1;ctb-1;mec-7::rfp* control line (Table S3, *isp-1(qm150);ctb-1(qm189);rwEx16[ceh-23+mec-7::rfp]*, line 1 versus *isp-1(qm150);ctb-1(qm189);rwEx18[mec-7::rfp]*, line 3). Thus, our data showed that overexpressing *ceh-23* can provoke a lifespan extension that is greater than that normally produced by overexpressing *ceh-23* in wild-type worms. This suggests that the effect of overexpressing CEH-23 on longevity can be enhanced in *isp-1;ctb-1* mutants. One intriguing hypothesis is that there is an optimal elevated level of *ceh-23* for maximal longevity increase that was not reached in wild-type worms overexpressing CEH-23. Since METC mutants already have elevated levels of *ceh-23*, the transgene introduced into *isp-1;ctb-1* mutants could promote further lifespan increase due to higher levels of *ceh-23*. We also noticed that worms overexpressing CEH-23 exhibited similar development rates and brood size as their respective control lines (unpublished data). Thus, CEH-23 plays a role in longevity determination that is independent of any obvious effects on the pace of development of the worms.

## Discussion

Signals from the mitochondrion to the nucleus are of crucial importance in the establishment of cellular adaptations resulting from altered METC function [5],[21]–[25],[50]. In yeast, mitochondrial dysfunction can induce nuclear expression changes that are key to lifespan determination through a signaling pathway that involves the RTG genes [21]. However, it is unknown whether similar retrograde signaling from the mitochondrion to the nucleus plays a role in longevity maintenance of animals. Through a targeted RNAi screen, we revealed three putative nuclear transcription factors that are broadly required for the longevity increase of METC mutants regardless of the specific lesions. Because Yang et al. recently showed that inactivation by RNAi or genomic mutation of the same subunit of the METC trigger different mechanisms of longevity [19], it will be interesting to test in the future whether any of the transcription factors identified in our screen as mediators of the long-lived phenotype of mitochondrial mutants will also be required for worms that live long due to mitochondrial dysfunction induced by RNAi knockdown.

Our findings implicate specific transcription factors in the extended longevity associated with mitochondrial mutations and strongly support the model that signaling from the mitochondria to the nucleus can modulate longevity in an animal. Our results nicely complement two recent *C. elegans* studies showing that mitochondrial perturbations are associated with major alterations in nuclear gene expression [48],[51]. While it is tempting to speculate that the transcription factor candidates we uncovered in this study might act to regulate the gene expression changes reported in the previous microarray studies, our data also highlight differential molecular outputs downstream of METC alterations. For instance, Cristina et al. proposed that changes in the expression levels of *fstr-1/2* contribute to the phenotypic outcomes of the *clk-1* mutation, as RNAi knockdown of *fstr-1/2* suppressed both the lifespan extension and the slow behaviors of *clk-1* mutant worms [48]. On the contrary, our data indicate that CEH-23 is specifically important for longevity but not the developmental phenotype of *isp-1* and *isp-1;ctb-1* mutants. Moreover, while *fstr-1/2* is uniquely required for the prolonged lifespan of *clk-1* mutant, but not that of *isp-1* mutant, we showed that *ceh-23* contributes to the extended longevity associated with *clk-1*, *isp-1*, *and isp-1;ctb-1* mutations, suggesting that the retrograde signaling pathway involving *ceh-23* can be triggered by different METC mutations regardless of the specific lesions. These observations are consistent with a model that *fstr-1/2* and *ceh-23* act in two different pathways to affect longevity. Along a similar line, the nuclear hormone receptor NHR-49 has recently been shown to mediate a number of compensatory metabolic responses upon METC dysfunction in *C. elegans*, but NHR-49 was not found to affect the longevity increase associated with METC impairment [52]. These findings together suggest a model, similar to that proposed in yeast [23], in which multiple distinct mitochondrial retrograde signaling pathways, each inducing specific responses, modulate worm lifespan and physiology upon mitochondrial dysfunction.

Our RNAi screen revealed additional interesting insights into the molecular mechanisms that enable mitochondria to modulate longevity. First, we found that the long-lived phenotype of the several mitochondrial mutants tested greatly depends on three candidate transcription factors (C52B9.2; NHR-25; and CEH-23) that appear to have no substantial effect on the lifespan of the short-lived METC mutant *mev-1*. A previous study has shown that long-lived and short-lived mitochondrial mutants share a number of common changes in their nuclear transcriptional profiles when compared to wild-type worms [51]. One possibility is that those common gene expression changes account for the phenotypes shared between long-lived and short-lived METC mutants, such as slow development rate. Another hypothesis is that mitochondrial perturbations induce overlapping nuclear gene expression changes in both the long-lived and the short-lived mutant worms to affect lifespan, but additional detrimental effects in the short-lived mitochondrial mutants, for instance highly elevated levels of ROS [18],[53], contribute to the opposite phenotypes observed. Since the candidate transcription factors we identified specifically suppress the long-lived phenotype of several mitochondrial mutants but did not cause further shortening of the *mev-1* mutant lifespan, we propose that distinct molecular mechanisms likely underlie the differential longevity phenotypes of METC mutants in *C. elegans*. Another interesting finding from the screen is that three out of the four candidate transcription factors (with the exception of *ceh-23*) we identified to be important for the *isp-1;ctb-1* mutant longevity also appeared to be required for the prolonged lifespan of the genetic mimic of caloric restriction, *eat-2*. Our findings are consistent with previous observations [40] and suggest that *eat-2* and METC mutants employ common as well as distinct mechanisms to modulate lifespan.

Our RNAi screen identified *ceh-23* as specifically important for the longevity of both *isp-1* and *isp-1;ctb-1* mutant worms and we confirmed our findings using a *ceh-23* genetic mutation. One puzzling observation is that knocking down *ceh-23* by mutation has a stronger suppressor effect on the longevity of the *isp-1* mutant than on that of the *isp-1;ctb-1* mutant, whereas *ceh-23* RNAi knock-down affected the longevity of both mutants to a similar extent. It is possible that compensatory responses take place over generations in the *isp-1;ctb-1* mutant to overcome the lack of *ceh-23* but not in the *isp-1* mutant. Our screen revealed that at least one transcription factor, *nhr-265*, is necessary for the extended longevity of *isp-1;ctb-1* but not for that of *isp-1*, again supporting possible differential responses in *isp-1* versus *isp-1;ctb-1* mutants.

An exciting observation with *ceh-23* is that it appears to specifically mediate the effect of reduced METC on longevity but not on development, brood size, or resistance to paraquat. The fact that *ceh-23* deletion suppressed the long lifespan of *isp-1* mutant worms without affecting their resistance to oxidative stress further supports the hypothesis that a general heightened response to ROS is not sufficient to explain the extended longevity of METC worm mutants. This finding corroborates with a recent study demonstrating that knockdown of superoxide dismutase activities does not suppress the long lifespan caused by reduced METC [54]. Importantly, while previous evidence has hinted at the possibility that slow physiological rates can be separated from the longevity phenotype in worms [6] and flies [14], our data point to a molecular determinant that responds to mitochondrial perturbations to promote longevity without affecting other pleiotropic phenotypes commonly associated with METC perturbations. This finding has important implications as it suggests the possibility of harnessing the beneficial effect on lifespan by altered METC without compromising the critical roles mitochondria have on essential physiological processes.

In addition to being specifically required for the extended lifespan effect of mitochondrial mutations, we showed that overexpression of *ceh-23* alone is sufficient to confer longevity increase. Moreover, our data suggest the extent of lifespan increase is proportional to the levels of overexpressed CEH-23 and that overexpression of CEH-23 increases lifespan without affecting developmental rate. Taken together, our data support CEH-23 to be a novel longevity determinant. *C. elegans ceh-23* represents a diverged homeobox protein that was first identified in a search for HOM-C genes [55]. Members of the homeobox-containing protein family generally have function related to transcription. In agreement with a putative role in transcription, we found that CEH-23 expression is nuclear in a set of neurons and the intestine. Not much is known about CEH-23 functions in *C. elegans*, other than its role in the transcriptional regulation of the development of several neurons where CEH-23 is expressed [42],[46],[56] and the fact that *ceh-23* is not required for longevity maintenance under normal conditions [43]. Our study is the first, to our knowledge, to suggest a role for CEH-23 in longevity. The homeobox of *C. elegans* CEH-23 presents some similarities with the *distal-less* and *empty spiracles* protein families, but clear orthologs of CEH-23 in other species have not been identified based on sequence comparison [42]. Nevertheless, functional homologs of CEH-23 likely exist, and in the future, it will be very interesting to examine possible roles of homeobox proteins in responding to mitochondrial dysfunction to modulate physiology and longevity in mammals.

It is intriguing that CEH-23 localization is confined to a subset of neurons, most of which are sensory neurons, and the intestine. Importantly, strains carrying the *gfp::ceh-23* transgene also exhibited increased lifespan when compared to control animals (unpublished data), suggesting that overexpression of *ceh-2*3 in a subset of neurons, most of which are sensory neurons, and/or the intestine is sufficient to modulate longevity. Interestingly, one of the sets of neurons expressing CEH-23 [42] is the ASI neurons, which are implicated in a model of caloric restriction-mediated longevity increase in *C. elegans* [57]. The restricted expression pattern of CEH-23 suggests that retrograde signaling from mitochondria to CEH-23 in specific neuron(s) and/or the intestine is sufficient to cause longevity changes. It is also possible that particular tissues, for instance highly ATP-dependent tissues like neurons, are more affected by METC mutations and are therefore key to mitochondrial signaling that modulates longevity. Supporting this hypothesis, neuronal-specific knockdown of METC subunits is sufficient to extend longevity in *Drosophila* [14]. An alternate but not mutually exclusive possibility is that CEH-23 acts in a select subset of cells to integrate signals from dysfunctional mitochondria from the whole worm and modulate longevity in a non-cell autonomous manner. If CEH-23 acts as a transcription factor, one or more of the CEH-23 transcriptional targets may constitute a signal that can travel through the worms to coordinate aging rate of the entire organism. Future investigation of CEH-23 downstream targets will go a long way in revealing the mechanism of action of CEH-23 in mediating the lifespan extension of METC mutants.

Our study revealed a novel longevity determinant that specifically responds to altered mitochondrial function to affect lifespan in a non-cell autonomous manner, pointing to a new paradigm of the molecular pathways that enable mitochondria to impact aging and age-dependent diseases in an animal.

### Experimental Procedures

#### *C. elegans* strains

Most of the strains used were obtained from the *Caenorhabditis* Genetic Center. *The gmIs18[ceh-23::gfp]* strain [45] was a kind gift from the Bargmann and Garriga laboratories. The *ceh-23(ms23);isp-1(qm150);ctb-1(qm189)*, *ceh-23(ms23);isp-1(qm150)*, and the *isp-1(qm150);ctb-1(qm189);gmIs18[ceh-23::gfp]* strains were constructed using standard genetic methods. All strains were out-crossed into wild-type N2 in our lab at least four times and cultured using standard methods [58].

### Generation of *ceh-23* Construct and Transgenic Lines

All constructs were verified by sequencing or restriction fragment length. Sequences of the primers used for PCR are available upon request. The genomic sequence of *ceh-23* and the transgenes are illustrated in Figure S2.

#### *ceh-23* transgene under control of endogenous *ceh-23* promoter

A 10.3 Kbp genomic fragment containing the 7.3 Kbp sequence upstream of the *ceh-23* coding region, the 1.8 Kbp *ceh-23* coding region, and the 1.1 Kbp sequence downstream of the *ceh-23* coding region was amplified from genomic DNA by PCR.

#### *ceh-23-GFP* transgene under control of endogenous *ceh-23* promoter

A GFP coding sequence without stop codon was amplified by PCR from the plasmid pPD95-81. The amplified GFP sequence was then fused in frame at the N-terminal of the *ceh-23* coding region into the 10.3 Kbp genomic fragment described above [59].

For generation of transgenic animals carrying extrachromosomal arrays of *ceh-23* or *gfp::ceh-23*, 10 ng/µl or 1 ng/µl of the *ceh-23* transgene (*rwEx16[ceh-23+mec-7::rfp]* and *rwEx17[ceh-23+mec-7::rfp]*, respectively) or 10 ng/µl of the *gfp::ceh-23* transgene (*rwEx19[gfp::ceh-23+mec-7::rfp]*) and 50 ng/µl of *mec7*::*rfp* as a co-injection marker and 50 ng/µl Bluescript plasmid filler DNA were microinjected into the gonads of adult wild-type animals using standard methods [60]. F1 progeny were selected on the basis of RFP fluorescence. Individual F2 worms were isolated to establish independent lines. The worms used as controls for the lifespan experiments were injected with 50 ng/µl of *mec7*::*rfp* as a co-injection marker and 60 ng/µl Bluescript plasmid filler DNA (*rwEx18[mec-7::rfp]*). An increased expression of *ceh-23* was detected by qRT-PCR for all the independent *ceh-23* transgenic lines used in the study (unpublished data). The *isp-1(qm150);ctb-1(qm189);rwEx16[ceh-23+mec-7::rfp]*, *isp-1(qm150);ctb-1(qm189);rwEx18[mec-7::rfp]*, *isp-1(qm150);ctb-1(qm189);rwEx19[gfp::ceh-23+mec-7::rfp]*, and *isp-1(qm150);rwEx19[gfp::ceh-23+mec-7::rfp]* strains were constructed following standard genetic methods. For one of these strains, *rwEx16[ceh-23::gfp+mec-7::rfp]* line 3, the extrachromosomal DNA spontaneously integrated into the genome, giving rise to the strain rw*Is21[ceh-23+mec-7::rfp]*. The control *rwIs19[mec-7::rfp]* strain was obtained by irradiation of *rwEx18[mec-7::rfp]* L4 worms with 1,500 rads gamma radiation and inspection of progeny for 100% transgene transmission. The *rwIs19[mec-7::rfp]* and *rwIs21[ceh-23+mec-7::rfp]* strains were out-crossed, respectively, once and three times into wild-type N2. The *ceh-23(ms23);isp-1(qm150);rwIs19[mec-7::rfp]* and *ceh-23(ms23);isp-1(qm150);rwIs21[ceh-23+mec-7::rfp]* strains were constructed using standard genetic procedure.

### RNAi Lifespan Screen

RNAi bacteria from the commercial *C. elegans* Transcription Factors Library (Gene Service Inc.) or HT115 carrying the empty vector RNAi L4440, *daf-16* RNAi clone, or *age-1* RNAi clone were grown in Luria broth with 50 µg/ml ampicillin at 37°C for 8–12 h and seeded onto 35 mm NGM plates containing 4 mM IPTG, and induced overnight at room temperature. Duplicate plates of each RNAi clones and quadruplicate plates of the empty vector L4440 control RNAi were used in each lifespan assay. Gravid *isp-1;ctb-1* mutant worms were allowed to lay ∼30 eggs by plate and the progeny grew on RNAi plates at 20°C until they developed into the young adult stage. The young adult worms were re-fed with 3-fold concentrated RNAi bacteria and 50 µg/ml of 5-fluoro-2′-deoxyuridine (FUDR) to prevent the growth of progeny. Starting at day 10 of adulthood, worms were scored every 2–3 d, and those that failed to respond to a gentle prodding with a platinum wire were scored as dead. Worms that died of protruding or bursting vulva or bagged were censored on the day of death. We routinely tested 96 RNAi clones in each independent experiment. Lifespan is defined as the time elapsed from when FUDR was added to the worms (day 0 of adult lifespan) until the worms are scored as dead. The survival function of each worm population was estimated using the Kaplan Meier estimator (SPSS software) and statistical analysis was done using log-rank test. *p*≤0.001 was considered as significantly different from the control population.

### Lifespan Assays

RNAi bacteria were freshly transformed before each trial and the bacteria were induced with 4 mM IPTG after reaching an optical density at 600 nm value of 0.8. In some experiments the worms were fed as needed by adding 150 µl bacteria solution to the plates. In some experiments, the worms were transferred every other day into plates seeded with freshly induced bacteria. Using a feeding or a transfer protocol did not significantly change the results. The worms were scored every 1 or 2 d. Each lifespan assay was tested on duplicate or triplicate plates in at least two independent experiments.

#### Lifespan assays with OP50 bacteria

The lifespan assays performed on wild-type worms, *ceh-23(ms23)* mutant, *isp-1(qm150)* mutants, *ceh-23(ms23);isp-1(qm150)* mutants, *isp-1(qm150);ctb-1(qm189)*, *ceh-23(m23);isp-1 (qm150);ctb-1(qm189)* mutants, and on transgenic worms were performed on OP50 bacteria. The optical density at 600 nm of the bacteria cultures was adjusted to the same value (1× bacteria: values at 0.175 for 1/10 dilution). Bacteria were seeded at 1× or 10× concentration (the concentration of the bacteria did not affect the differences between the lifespan of the different strains tested). Worms were transferred onto fresh plates every other day and scored as described above. Each lifespan assay was tested on in duplicate or triplicate plates in at least three independent experiments. For the lifespan assays involving transgenic worms, transgenic parents (based on RFP fluorescence) were used for the egg lay. At day 0 of adulthood, 30 transgenic worms (based on RFP fluorescence) from the progeny were transferred onto fresh plates.

### Statistical Analysis of the Lifespan Assays

The independent trials were either analyzed separately as described above or, when appropriate, the data from the independent trials were pooled and compared using stratified log rank test after testing homogeneity among strata (individually controlled experimental replicates). In some cases, the mean variation in lifespan for each strain tested was calculated and compared to wild-type worms for each experiment. The averaged mean variations were analyzed using mixed model analysis with *ceh-23* mutation as the fixed effect and experiment as the random effect.

### Assays for Developmental Rate and Brood Size Phenotypes

Developmental rate and brood size were monitored as previously described [61]. Worms were grown on plates seeded either with OP50 or with RNAi bacteria induced with 4 mM IPTG. *Self-brood size:* Young adults were singled onto fresh plates incubated at 20°C and transferred onto fresh plates every 12 h to prevent overcrowding until egg laying ceased. The progeny produced on each plate was counted 36 h after removal of the parent. The mean self-brood size obtained for worms of each strain was compared using Student's *t* test. *Time to reach adulthood*: 60 synchronized eggs were plated and incubated at 20°C until they reached L4 stage and then scored for vulva formation every 6–8 h. The time elapsed from the egg stage until the stage of complete vulva formation was considered as the time necessary to reach adulthood. The mean number of hours required to reach adulthood obtained for worms of each strain was compared using Student's *t* test. Similar results were obtained when animals were scored for adult alae appearance.

### Paraquat Assays

Thirty worms synchronized at the L4 stage or at the first day of adulthood or at day 4 of adulthood were transferred into plates seeded with OP50 bacteria and containing 16 mM paraquat and 50 µg/ml of 5-fluoro-2′-deoxyuridine (FUDR). Worms were grown at 25°C for 3 d (to avoid vulva bursting) and then transferred to 20°C. Worms were scored every day. Survival on paraquat was defined as the time elapsed from when paraquat was added until the worms are scored as dead. The survival function of each worm population was estimated using the Kaplan Meier estimator (SPSS software), and statistical analysis was done using log rank test. *p*≤0.001 was considered as significantly different from the control population.

### RNA Isolation and Quantitative Reverse Transcription PCR (qRT-PCR)

Synchronized populations of ∼4,000 eggs for each strain tested were either grown to late L4 staged worms (confirmed by Normaski images of the gonad arms) at 20°C and harvested or grown to the young adult stage, treated with FUDR, and harvested 4 d later. Total RNA was isolated using Tri-reagent (Molecular Research Center, Inc.) [62]. cDNAs were synthesized with random hexamers using SuperScript III First-Strand Kit (Invitrogen). qRT-PCR reactions were performed using iQ SYBR Green Supermix (BIO-RAD) and the MyiQ Single Color Real-Time PCR Detection System (BIO-RAD). Melting curve analysis was performed for each primer set at the end to ensure the specificity of the amplified product. *act-1* was used as the internal control so that the RNA level of each gene of interest was normalized to the level of *act-1*. As an additional control, in each experiment, mRNA expression of *tbb-2* was measured to ensure that *tbb-2* expression normalized to *act-1* did not change between the strains tested. The qRT-PCR experiments were repeated at least three times using independent RNA/cDNA preparations. Quantitative PCR primer sequences are available upon request.

### GFP Localization and Quantification

For GFP localization, worms at L1–L2 stage were paralyzed with levamisole on an agar pad. The GFP and RFP expressions were visualized at 60× magnification using a Leica DM 5000B microscope. Images were captured using OpenLab software. For GFP quantification, worms at L1–L2 or L4 stage were paralyzed with levamisole on an agar pad. The GFP expression was visualized at 60× magnification using a Leica SP2 confocal microscope. Images were acquired using Leica software. In the GFP channel, images were collected using a *z*-stack acquisition at 1 µm step interval, with each frame averaged 4 times, and projected in a 20 µm *z*-stack covering the entire worm. Some of the experiments were performed as double-blind assays. The mean GFP intensities were quantified using Image J and compared between strains using Student's *t* test.

*Note:* While this paper was in review, several studies were published [63]–[65] that greatly advanced our understanding of the molecular mechanisms by which METC mutations prolong lifespan in *C. elegans*. Future investigations of how CEH-23 integrates with the new pathways revealed by these studies will provide important new insights into how mitochondria impact animal physiology and longevity.

## Supporting Information

Figure S1 Targeted RNAi screen strategy to identify putative transcription factors that specifically suppress the long-lived phenotype of mitochondrial mutant worms. Using the *C. elegans* Transcription Factor RNAi Library (Gene Service Inc.) and feeding RNAi, we identified CEH-23 as a specific mediator of the longevity phenotype of *isp-1;ctb-1*, *isp-1*, and *clk-1* mutant worms.
(EPS)

Figure S2 Schematic of *ceh-23* (worm base). Exons are shown in blue and untranslated regions in gray. *ZK652.6* and *ZK652.8* are, respectively, the genes upstream and downstream of *ceh-23*. Dotted lines indicate DNA segments used to generate the four RNAi constructs (*ceh-23A*, *ceh-23B*, *ceh-23C*, and *ceh-23 ahringher* constructs) used in this study. The *ms23* deletion that covers half of the predicted homeobox of *ceh-23* is shown in red. The solid black lines indicate the segment of DNA that was amplified by PCR for injection to generate *rwEx16[ceh-23+mec-7::rfp]*, *rwEx17[ceh-23+mec-7::rfp]*, and *rwEx19[gfp::ceh-23+mec-7::rfp]* transgenic worms. The insertion site of the GFP sequence in the transgene is also indicated.
(EPS)

Figure S3 Effects of three *ceh-23* RNAi clones on the lifespan of wild-type and mitochondrial mutant worms. The data presented are from two pooled independent experiments. (A) Wild-type worms treated with *ceh-23* RNAi construct A (blue curve) or *ceh-23* RNAi construct B (red curve) or *ceh-23* RNAi construct C (green curve) exhibited the same lifespan as wild-type worms treated with empty vector control (black curve). (B) *clk-1(e2519)* worms treated with *ceh-23* RNAi construct A (blue curve) or *ceh-23* RNAi construct B (red curve) or *ceh-23* RNAi construct C (green curve) exhibited a lifespan shorter than that of *clk-1(e2519)* worms treated with empty vector control (black curve). (C) *isp-1(qm150);ctb-1(qm189)* worms treated with *ceh-23* RNAi construct A (blue curve) or *ceh-23* RNAi construct B (red curve) or *ceh-23* RNAi construct C (green curve) exhibited a lifespan shorter than that of *isp-1(qm150);ctb-1(qm189)* worms treated with empty vector control (black curve). (D) *isp-1(qm150)* worms treated with *ceh-23* RNAi construct A (blue curve) or *ceh-23* RNAi construct B (red curve) or *ceh-23* RNAi construct C (green curve) exhibited a lifespan shorter than that of *isp-1(qm150)* worms treated with empty vector control (black curve).
(EPS)

Figure S4 Survival on paraquat. The data presented are from two pooled independent experiments. (A) *isp-1* (red curve, mean survival 3.85±0.10 d) and *ceh-23;isp-1* (green curve, mean survival 4.13±0.13 d) mutants exhibited a better mean survival than wild-type (black curve, mean survival 2.48±0.08 d) and *ceh-23* mutants (blue curve, mean survival 2.58±0.06 d) when paraquat treatment was initiated at L4 stage. (B) Wild-type (black curve, mean survival 4.22±0.11 d), *ceh-23* mutant (blue curve, mean survival 4.10±0.18 d), *isp-1* (red curve, mean survival 4.94±0.23 d), and *ceh-23;isp-1* (green curve, mean survival 5.00±0.19 d) mutant worms exhibited the same mean survival when paraquat treatment was initiated at day 0 of adulthood. (C) Wild-type (black curve, mean survival 7.90±0.25 d), *ceh-23* mutant (blue curve, mean survival 7.71±0.29 d), *isp-1* (red curve, mean survival 7.94±0.26 d), and *ceh-23;isp-1* (green curve, mean survival 8.44±0.28 d) mutant worms exhibited the same mean survival when paraquat treatment was initiated at day 4 of adulthood. (D) Mean survival of wild-type (black), *ceh-23* mutant (blue), *isp-1* (red), and *ceh-23;isp-1* (green) mutant worms exposed to paraquat starting at stage L4, day 0, or day 4 of adulthood.
(EPS)

Figure S5 *ceh-23* expression is elevated in mitochondrial mutants. (A) GFP expression in *gmls18[ceh-23::gfp]* and in *isp-1;ctb-1;gmls18[ceh-23::gfp]* transgenic mutant worms at L1 stage was visualized using confocal microscopy. CEH-23::GFP expression is shown in green and fluorescent image was merged with DIC image. The worms were mounted on the same agar pad. (B) Quantification of the GFP expression levels in the *gmls18[ceh-23::gfp]* and in *isp-1;ctb-1;gmls18[ceh-23::gfp]* transgenic mutant worms shown in (A).
(EPS)

Figure S6 The transgene overexpressing *ceh-23* is functional. (A) *rwEx16[ceh-23+mec-7::rfp]* worms exhibited a longer mean lifespan than control *rwEx18[mec-7::rfp]* worms (black line) when treated with empty vector (red line), but had a lifespan similar to control *rwEx18[mec-7::rfp]* worms when treated with *ceh-23* RNAi (blue line). (B) *rwIs21[ceh-23+mec-7::rfp]* worms (red line) exhibited the same mean lifespan as wild-type worms (black curve). *ceh-23(ms23);isp-1(qm150);rwIs21[ceh-23+mec-7::rfp]* worms (green curve) exhibited a longer mean lifespan than *ceh-23(ms23);isp-1(qm150);rwIs19[mec-7::rfp]* control worms (blue curve). Quantitative data are included in Table S3.
(EPS)

Table S1 Quantitative data and statistical analyses of mean adult lifespan presented in Figure 1 and Tables 1 and 2.
(PDF)

Table S2 Quantitative data and statistical analyses of adult lifespan of wild-type, *ceh-23*, *isp-1;ctb-1*, *ceh-23;isp-1;ctb-1*, *isp-1*, and *ceh-23;isp-1* mutant worms. The data presented in Figure 2 are from Experiments 2 and 6. We tested up to six individuals' genetic isolates of *isp-1;ctb-1;ceh-23* mutant worms and five individuals genetic isolates of *ceh-23;isp-1* mutant worms.
(PDF)

Table S3 Quantitative data and statistical analyses of adult lifespan of *rwEx16[ceh-23+mec-7::rfp]* (injected with 10 ng/µl), *rwEx17[ceh-23+mec-7::rfp]* (injected with 1 ng/µl), *rwEx18[mec-7::rfp]*, *isp-1(qm150);ctb-1(qm189);rwEx16[ceh-23+mec-7::rfp]*, *isp-1(qm150);ctb-1(qm189);rwEx18[mec-7::rfp]*, *rwIs21[ceh-23+mec-7::rfp]*, *ceh-23(ms23);isp-1(qm150);rwIs21[ceh-23+mec-7::rfp]*, and *ceh-23(ms23);isp-1(qm150);rwIs19[mec-7::rfp]* (see Experimental Procedures). The experiments presented in Figure 4 are from Experiments 1 and 5.
(PDF)

## Abbreviations

AHA: aryl hydrocarbon receptor associated protein
ASI: amphid sensilla
ATP: adenosine triphosphate
BLAST: basic local alignment search tool
*C. elegans*: *Caenorhabditis elegans*
CAN: canal associated nerve
CEH: *C. elegans* homeobox
CEP: *C. elegans* P53 like protein
CLK: clock (biological timing) abnormality
CTB: cytochrome b
DAF: abnormal dauer formation
DVE: defective proventriculus homolog
EAT: eating: abnormal pharyngeal pumping
FOXO: forkhead box O
FSTR: faster
GFP: green fluorescent protein
HOM-C: cluster homeotic genes
HSF: heat shock protein
IIS: insulin/insulin like growth factor signaling
ISP: iron sulfur protein
LIN: abnormal cell lineage
METC: mitochondrial electron transport chain
MEV: abnormal methyl viologen sensitivity
mRNA: messenger RNA
NHR: nuclear hormone protein
PHA: defective pharynx development
qRT-PCR: quantitative reverse transcription PCR
RNAi: RNA interference
ROS: Reactive Oxygen Species
RTG: retrograde genes
UTR: untranslated region
