# Supplementary material for: The Homeobox Protein CEH-23 Mediates Prolonged Longevity in Response to Impaired Mitochondrial Electron Transport Chain in C. elegans
Source: PLoS Biol. 2011 Jun 21;9(6):e1001084. doi: 10.1371/journal.pbio.1001084 (PMC3119657; doi:10.1371/journal.pbio.1001084)
Supplement: Table S1 — Quantitative data and statistical analyses of mean adult lifespan presented in Figure 1 and Tables 1 and 2. (PDF) [file pbio.1001084.s007.pdf]

Table S1: Quantitative data and statistical analyses of mean adult lifespan presented in Figure 1 and Tables 1 and 2.

Strain	Mean adulthood lifespan +/- s.d (days)	Censored worms (%)	n	Mean adulthood lifespan +/- s.d (days)	censored worms (%)	n	p-value (stratified log-rank test) compared to control RNAi	Number of experiments
	Control RNAi			***dve-1*** RNAi				
isp-1 (qm150); ctb-1 (qm189)	27.05 +/- 0.34	13.8	355	12.02 +/- 0.24	33.5	266	0.000	3
Wild-type	19.50 +/- 0.28	17.2	186	9.07 +/- 0.17	43.5	214	0.000	2
daf-16 (mgDf47)	11.45 +/- 0.20	8.7	69	7.47 +/- 0.19	9.7	62	0.000	1
age-1 (hx546)	25.62 +/- 0.72	28.2	85	12.09 +/- 0.38	47.5	80	0.000	1
	Control RNAi			***lin-40*** RNAi				
isp-1 (qm150); ctb-1 (qm189)	25.99 +/- 0.29	8.8	330	13.62 +/- 0.41	22.5	271	0.000	3
Wild-type	18.90 +/- 0.20	3.9	255	15.55 +/- 0.36	28.1	128	0.000	2
daf-16 (mgDf47)	12.51 +/- 0.16	3.3	90	10.29 +/- 0.15	12.7	102	0.000	2
age-1	32.38	7.8	64	14.02	6.1	49	0.000	2

(hx546)	+/- 0.84			+/- 0.47				
	Control RNAi			***nhr-49*** RNAi				
isp-1 (qm150); ctb-1 (qm189)	27.57 +/- 0.33	4.0	225	14.91 +/- 0.20	4.1	172	0.000	3
Wild-type	23.15 +/- 0.33	5.3	75	10.29 +/- 0.25	0.0	81	0.000	2
daf-16 (mgDf47)	11.29 +/- 0.20	7.9	63	7.00 +/- 0.21	0.0	88	0.000	2
age-1 (hx546)	24.12 +/- 0.59	14.3	147	16.49 +/- 0.63	35.8	187	0.000	3
	Control RNAi			***C52B9.2*** RNAi				
isp-1 (qm150); ctb-1 (qm189)	26.39 +/- 0.26	13.8	516	16.32 +/- 0.16	5.3	339	0.000	4
Wild-type	18.77 +/- 0.20	10.9	350	14.54 +/- 0.11	3.2	309	0.000	3
daf-16 (mgDf47)	11.62 +/- 0.13	6.0	149	10.22 +/- 0.12	1.2	169	0.000	3
age-1 (hx546)	24.59 +/- 0.55	18.8	149	23.13 +/- 0.46	6.3	158	0.026	3
isp-1 (qm150)	22.28 +/- 0.62	20.9	201	15.60 +/- 0.46	3.8	106	0.000	2
clk-1 (e2519)	22.83 +/- 0.22	0.0	178	14.85 +/- 0.16	0.0	143	0.000	2
mev-1 (kn1)	12.07 +/- 0.19	9.0	246	10.62 +/- 0.23	10.7	221	0.000	2
eat-2 (ad1116)	22.75 +/- 0.46	1.7	121	15.95 +/- 0.27	0	123	0.000	2
	Control RNAi			***ZC123.3*** RNAi				
isp-1 (qm150);	28.29 +/-	3.9	389	17.75 +/-	6.8	322	0.000	3

ctb-1 (qm189)	0.27			0.36				
Wild-type	20.43 +/- 0.25	4.4	91	11.25 +/- 0.28	65.9	88	0.000	2
daf-16 (mgDf47)	12.51 +/- 0.16	3.3	90	11.01 +/- 0.41	50.0	84	0.000	2
age-1 (hx546)	24.65 +/- 0.85	20.5	83	21.24 +/- 1.02	65.6	61	0.014	2
	Control RNAi			***ceh-20*** RNAi				
isp-1 (qm150); ctb-1 (qm189)	24.69 +/- 0.41	7.7	169	16.77 +/- 0.78	35.7	157	0.000	3
Wild-type	18.47 +/- 0.45	2.1	94	16.23 +/- 0.34	32.4	145	0.000	2
daf-16 (mgDf47)	11.45 +/- 0.20	8.7	69	10.33 +/- 0.23	24.7	77	0.001	2
age-1 (hx546)	25.62 +/- 0.72	28.2	85	19.78 +/- 1.03	61.8	68	0.000	2
	Control RNAi			***nhr-25*** RNAi				
isp-1 (qm150); ctb-1 (qm189)	26.12 +/- 0.28	8.5	539	17.74 +/- 0.40	13.1	275	0.000	5
Wild-type	19.45 +/- 0.16	0.9	349	17.91 +/- 0.26	17.4	268	0.000	4
daf-16 (mgDf47)	10.57 +/- 0.37	0.0	70	9.68 +/- 0.33	20.8	72	0.043	2
age-1 (hx546)	23.02 +/- 0.49	3.1	129	26.16 +/- 1.48	18.0	50	0.001	2
isp-1 (qm150)	24.43 +/- 0.49	19.6	135	13.04 +/- 0.52	17.7	102	0.000	2
clk-1 (e2519)	27.01 +/- 0.41	0.0	114	19.08 +/- 0.53	21.8	110	0.000	1

mev-1 (kn1)	13.86 +/- 0.23	0.5	211	12.84 +/- 0.27	7.9	152	0.007	2
eat-2 (ad1116)	24.28 +/- 0.35	5.7	106	13.57 +/- 0.75	25.2	115	0.000	1
	Control RNAi			***ceh-23*** RNAi				
isp-1 (qm150); ctb-1 (qm189)	25.62 +/- 0.30	5.8	308	18.26 +/- 0.27	7.1	210	0.000	3
Wild-type	18.90 +/- 0.20	3.9	255	21.14 +/- 0.17	0.6	158	0.000	3
daf-16 (mgDf47)	12.15 +/- 0.12	3.5	170	14.91 +/- 0.19	2.6	152	0.000	3
age-1 (hx546)	24.12 +/- 0.59	14.3	147	27.24 +/- 0.50	0.0	111	0.007	3
isp-1 (qm150)	22.28 +/- 0.62	20.9	201	15.80 +/- 0.34	3.4	89	0.000	3
clk-1 (e2519)	22.83 +/- 0.22	0.0	178	15.55 +/- 0.28	2.6	115	0.000	3
mev-1 (kn1)	13.70 +/- 0.19	0.9	110	14.04 +/- 0.15	6.5	108	0.085	2
eat-2 (ad1116)	27.70 +/- 0.23	8.6	185	27.73 +/- 0.20	8.2	195	0.698	2
	Control RNAi			***nhr-119*** RNAi				
isp-1 (qm150); ctb-1 (qm189)	26.24 +/- 0.31	3.4	291	22.32 +/- 0.34	2.8	217	0.000	3
Wild-type	20.41 +/- 0.30	2.6	153	18.45 +/- 0.23	0.6	178	0.000	3
daf-16 (mgDf47)	11.22 +/- 0.18	7.1	70	10.92 +/- 0.10	3.2	126	0.053	2
age-1 (hx546)	26.06 +/-	4.7	193	25.54 +/-	10.0	150	0.373	2

	0.53			0.57				
	Control RNAi			***nhr-265*** RNAi				
isp-1 (qm150); ctb-1 (qm189)	26.09 +/- 0.32	6.6	271	23.02 +/- 0.31	4.6	303	0.000	4
Wild-type	19.40 +/- 0.18	1.1	353	20.33 +/- 0.16	4.0	351	0.000	5
daf-16 (mgDf47)	11.58 +/- 0.12	5.3	152	11.02 +/- 0.13	4.9	122	0.002	3
age-1 (hx546)	26.93 +/- 0.43	4.0	297	28.36 +/- 0.63	7.4	215	0.003	3
isp-1 (qm150)	25.53 +/- 1.45	1.9	53	23.50 +/- 1.18	12.5	40	0.022	2
clk-1 (e2519)	23.72 +/- 0.31	0.0	117	24.48 +/- 0.35	0.9	107	0.062	2
mev-1 (kn1)	13.86 +/- 0.23	0.5	211	14.68 +/- 0.20	2.2	224	0.011	2
eat-2 (ad1116)	21.53 +/- 0.24	23.3	148	20.69 +/- 0.25	0.6	178	0.025	2
	Control RNAi			***ceh-37*** RNAi				
isp-1 (qm150); ctb-1 (qm189)	25.86 +/- 0.30	11.6	415	22.82 +/- 0.32	5.7	385	0.000	4
Wild-type	18.55 +/- 0.17	5.4	350	17.06 +/- 0.20	5.0	221	0.000	3
daf-16 (mgDf47)	11.69 +/- 0.14	3.1	96	11.88 +/- 0.17	3.1	96	0.288	2
age-1 (hx546)	25.49 +/- 0.45	3.0	233	27.63 +/- 0.51	0.0	123	0.062	3
	Control RNAi			***lin-11*** RNAi				
isp-1 (qm150); ctb-1	26.83 +/- 0.27	12.3	635	23.77 +/- 0.29	7.7	467	0.000	7

(qm189)								
Wild-type	19.52 +/- 0.21	1.7	287	17.78 +/- 0.18	6.4	280	0.000	5
daf-16 (mgDf47)	11.54 +/- 0.12	5.0	159	11.04 +/- 0.09	4.1	270	0.001	3
age-1 (hx546)	25.49 +/- 0.45	3.0	233	20.98 +/- 0.57	12.9	140	0.000	3
	Control RNAi			***aha-1*** RNAi				
isp-1 (qm150); ctb-1 (qm189)	26.32 +/- 0.23	9.2	736	23.41 +/- 0.23	7.5	682	0.000	8
Wild-type	20.40 +/- 0.15	3.9	510	18.36 +/- 0.14	5.0	537	0.000	7
daf-16 (mgDf47)	11.09 +/- 0.09	4.1	320	10.57 +/- 0.10	4.4	227	0.001	3
age-1 (hx546)	26.03 +/- 0.47	18.1	282	27.06 +/- 0.58	22.4	250	0.073	5
	Control RNAi			***nhr-77*** RNAi				
isp-1 (qm150); ctb-1 (qm189)	26.91 +/- 0.29	4.4	297	24.06 +/- 0.35	3.8	182	0.000	3
Wild-type	21.52 +/- 0.19	5.1	254	19.91 +/- 0.22	4.3	210	0.000	3
daf-16 (mgDf47)	11.74 +/- 0.11	4.3	234	10.58 +/- 0.12	3.6	195	0.000	3
age-1 (hx546)	28.38 +/- 0.56	14.8	189	24.17 +/- 0.63	15.3	118	0.000	3
