# Supplementary material for: The Homeobox Protein CEH-23 Mediates Prolonged Longevity in Response to Impaired Mitochondrial Electron Transport Chain in C. elegans
Source: PLoS Biol. 2011 Jun 21;9(6):e1001084. doi: 10.1371/journal.pbio.1001084 (PMC3119657; doi:10.1371/journal.pbio.1001084)
Supplement: Table S2 — Quantitative data and statistical analyses of adult lifespan of wild-type, ceh-23, isp-1;ctb-1, ceh-23;isp-1;ctb-1, isp-1, and ceh-23;isp-1 mutant worms. The data presented in Figure 2 are from Experiments 2 and 6. We tested up to six individuals' genetic isolates of isp-1;ctb-1;ceh-23 mutant worms and five individuals genetic isolates of ceh-23;isp-1 mutant worms. (PDF) [file pbio.1001084.s008.pdf]

Table S2: Quantitative data and statistical analyses of adult lifespan of wild-type, *ceh-23*, *isp-1; ctb-1, ceh-23;isp-1;ctb-1, isp-1 and ceh-23;isp-1* mutant worms on 1X and 10X concentrated OP50 bacteria (see Material and Methods). The data presented in Figure 2 are from Experiments 2 and 6. We tested up to six individuals genetic isolates of *ceh-23*;*isp-1;ctb-1* mutant worms and five individuals genetic isolates of *ceh-23;isp-1* mutant worms.

Strain	Mean adulthood lifespan (days)	+/- s.d (days)	censored worms (%)	n	p-value (stratified log-rank test) compared to wild-type worms	p-value (stratified log-rank test) compared to *isp-1;ctb-1* worms	p-value (stratified log-rank test) compared to *isp-1* mutant worms
Experiment 1 OP50-1X							
Wild-type	20.49	0.28	0	128			
ceh-23	20.91	0.26	3	107	0.584		
isp-1;ctb-1	27.23	0.71	0	73	0.000		
ceh-23;isp-1;ctb-1 Isolate 1	26.63	0.38	13	104		0.006	
ceh-23;isp-1;ctb-1 isolate 2	25.57	0.43	8	83		0.000	
ceh-23;isp-1;ctb-1 isolate 3	26.79	0.51	0	113		0.180	
Experiment 2 OP50-1X							
Wild-type	17.78	0.22	1	116			
ceh-23	17.98	0.23	0	125	0.356		
isp-1;ctb-1	25.98	0.55	0	121	0.000		
ceh-23;isp-1;ctb-1 isolate1	23.92	0.48	0	118		0.001	
ceh-23;isp-1;ctb-1 isolate 2	24.06	0.43	0	128		0.000	
ceh-23;isp-1;ctb-1 isolate 3	23.40	0.49	2	113		0.000	
ceh-23;isp-1;ctb-1 isolate 4	23.37	0.44	2	110		0.000	
ceh-23;isp-1;ctb-1 isolate 5	23.23	0.44	0	120		0.000	
ceh-23;isp-1;ctb-1 isolate 6	23.34	0.46	0	117		0.000	
Experiment 3 OP50-1X							
Wild-type	19.28	0.36	16	74			
ceh-23	20.16	0.33	7	70	0.097		

isp-1;ctb-1	29.63	0.72	11	73	0.000		
ceh-23;isp-1;ctb-1 isolate1	29.32	0.44	1	70		0.094	
ceh-23;isp-1;ctb-1 isolate 2	27.45	0.66	3	69		0.008	
ceh-23;isp-1;ctb-1 isolate 5	29.28	0.66	16	74		0.394	
Experiment 3 OP50-10X							
Wild-type	17.49	0.32	12	60			
ceh-23	18.12	0.30	11	64	0.155		
isp-1;ctb-1	23.98	0.63	15	67	0.000		
ceh-23;isp-1;ctb-1 isolate1	23.63	0.56	4	56		0.234	
ceh-23;isp-1;ctb-1 isolate 2	24.40	0.71	12	49		0.713	
ceh-23;isp-1;ctb-1 isolate 5	20.74	0.57	8	68		0.000	
Experiment 4 OP50-10X							
Wild-type	17.09	0.22	18	73			
ceh-23	16.13	0.23	0	61	0.005		
isp-1;ctb-1	21.74	0.75	24	66	0.000		
ceh-23;isp-1;ctb-1 isolate1	19.12	0.43	15	71		0.000	
ceh-23;isp-1;ctb-1 isolate 2	19.25	0.77	25	64		0.001	
ceh-23;isp-1;ctb-1 isolate 5	18.69	0.37	4	67		0.020	
Experiment 5 OP50-1X							
Wild-type	17.79	0.24	7	150			
ceh-23	19.16	0.27	10	159	0.000		
isp-1	25.43	0.71	1	95	0.000		
ceh-23;isp-1 isolate1	23.31	0.74	3	77			0.029
ceh-23;isp-1 isolate 2	21.40	1.22	0	30			0.004
ceh-23;isp-1 isolate 3	20.66	0.62	0	62			0.000
ceh-23;isp-1 isolate 4	20.00	0.66	15	41			0.000
ceh-23;isp-1 isolate 5	21.07	0.77	7	41			0.000

Experiment 6 OP50-1X							
Wild-type	18.72	0.38	7	151			
ceh-23	18.57	0.35	0	82	0.057		
isp-1	25.50	1.80	0	14	0.000		
ceh-23;isp-1 isolate1	20.40	0.80	5	87			0.015
ceh-23;isp-1 isolate 2	19.04	1.09	0	45			0.002
ceh-23;isp-1 isolate 3	18.76	0.95	2	79			0.015
ceh-23;isp-1 isolate 4	17.86	1.61	3	31			0.002
ceh-23;isp-1 isolate 5	21.24	1.17	0	41			0.050
Experiment 7 OP50-1X							
Wild-type	16.76	0.26	1	93			
ceh-23	19.70	0.30	0	73	0.000		
isp-1	25.15	0.85	2	79	0.000		
ceh-23;isp-1 isolate 3	23.95	0.69	3	75			0.030
ceh-23;isp-1 isolate 3	22.57	1.56	15	34			0.073
Experiment 7 OP50-10X							
Wild-type	15.61	0.27	9	53			
ceh-23	15.40	0.27	8	78	0.752		
isp-1	19.63	0.73	6	81	0.000		
ceh-23;isp-1 isolate 4	19.01	0.98	5	35			0.398
ceh-23;isp-1 isolate 3	18.47	0.59	13	104			0.043
