# Supplementary material for: The Homeobox Protein CEH-23 Mediates Prolonged Longevity in Response to Impaired Mitochondrial Electron Transport Chain in C. elegans
Source: PLoS Biol. 2011 Jun 21;9(6):e1001084. doi: 10.1371/journal.pbio.1001084 (PMC3119657; doi:10.1371/journal.pbio.1001084)
Supplement: Table S3 — Quantitative data and statistical analyses of adult lifespan of rwEx16[ceh-23+mec-7::rfp] (injected with 10 ng/µl), rwEx17[ceh-23+mec-7::rfp] (injected with 1 ng/µl), rwEx18[mec-7::rfp], isp-1(qm150);ctb-1(qm189);rwEx16[ceh-23+mec-7::rfp], isp-1(qm150);ctb-1(qm189);rwEx18[mec-7::rfp], rwIs21[ceh-23+mec-7::rfp], ceh-23(ms23);isp-1(qm150);rwIs21[ceh-23+mec-7::rfp], and ceh-23(ms23);isp-1(qm150);rwIs19[mec-7::rfp] (see Experimental Procedures). The experiments presented in Figure 4 are from Experiments 1 and 5. (PDF) [file pbio.1001084.s009.pdf]

Table S3: Quantitative data and statistical analyses of adult lifespan of *rwEx16[ceh-23+mec-7::rfp]* (injected with 10ng/µl), *rwEx17[ceh-23+mec-7::rfp]* (injected with 1ng/µl), *rwEx18[mec-7::rfp]*, *isp-1(qm150);ctb-1(qm189);rwEx16[ceh-23+mec-7::rfp]*, *isp-1(qm150);ctb-1(qm189); rwEx18[mec-7::rfp]*, *rwIs21[ceh-23+mec-7::rfp]*, *ceh-23(ms23);isp-1(qm150);rwIs21[ceh-23+mec-7::rfp]* and *ceh-23(ms23);isp-1(qm150);rwIs19[mec-7::rfp]* (see Experimental Procedures). The experiments presented in Figure 4 are from experiments 1 and 5.

**Strain**	**Mean adulthood lifespan (days)**	**+/- s.d (days)**	**censored worms (%)**	**n**	**p-value (stratified log-rank test) compared to control *rwEx18[mec-7::rfp]*, line 3 in corresponding background**
**Wild-Type background**					
Experiment 1					
Control rwEx18[mec-7::rfp]					
Line 1	16.53	0.33	18	50	
Line 2	16.11	0.33	11	63	
Line 3	16.11	0.27	2	90	
rwEx17[ceh-23+mec-7::rfp] (1ng/µl)					
Line 1	16.90	0.42	0	42	0.022
Line 2	16.95	0.23	0	80	0.036
rwEx16[ceh-23+mec-7::rfp] (10ng/µl)					
Line 1	18.02	0.44	8	66	0.000
Line 2	19.27	0.38	0	75	0.000
Line 3	19.39	0.38	3	77	0.000
Line 4	19.87	0.53	0	52	0.000
Experiment 2					
Control rwEx18[mec-7::rfp]					
Line 1	16.25	0.28	1	84	
Line 2	16.55	0.24	0	66	
Line 3	15.60	0.70	0	20	
rwEx17[ceh-23+mec-7::rfp] (1ng/µl)					
Line 1	16.99	0.29	4	71	0.060
Line 2	15.75	0.19	2	87	0.590
Line 3	16.27	0.43	6	16	0.686
rwEx16[ceh-23+mec-7::rfp] (10ng/µl)					
Line 1	16.75	0.20	0	80	0.070
Line 2	16.66	0.18	1	89	0.093
Line 3	16.77	0.20	3	61	0.071
Line 4	18.69	0.30	0	98	0.000

Experiment 3					
Control rwEx18[mec-7::rfp]					
Line 1	14.30	0.22	4	122	
Line 3	14.23	0.28	7	115	
rwEx16[ceh-23+mec-7::rfp] (10ng/µl)					
Line 4	16.53	0.34	1	92	0.000
Line 5	16.59	0.27	18	51	0.000
Line 6	16.06	0.23	13	121	0.000
Line 7	15.07	0.24	9	99	0.107
Experiment 4					
Control rwEx18[mec-7::rfp]					
Line 3	14.89	0.17	6	107	
rwEx16[ceh-23+mec-7::rfp] (10ng/µl)					
Line 1	15.88	0.16	4	92	0.000
Line 4	16.47	0.32	0	110	0.000
Experiment 5					
Control rwEx18[mec-7::rfp]					
Line 3	16.84	0.29	3	74	
rwEx16[ceh-23+mec-7::rfp] (10ng/µl)					
Line 1	17.84	0.31	4	103	0.022
Line 3	18.44	0.32	6	99	0.000
Line 4	18.28	0.31	3	117	0.001
Line 5	21.08	0.44	10	95	0.000
Experiment 6					
Control rwEx18[mec-7::rfp]					
Line 3	18.72	0.33	12	107	
rwEx16[ceh-23+mec-7::rfp] (10ng/µl)					
Line 1	17.84	0.25	6	121	0.021
Line 3	17.97	0.31	11	119	0.118
Line 4	19.67	0.39	2	99	0.019
Line 5	21.66	0.33	12	119	0.000
***isp-1;ctb-1* background**					
Experiment 4					
Control isp-1(qm150);ctb-1(qm189);rwEx18[mec-7::rfp]					
Line 3	17.73	0.30	7	106	
isp-1(qm150);ctb-1(qm189);rwEx16[ceh-23+mec-7::rfp]					
Line 1	16.40	0.32	5	120	0.005
Line 4	19.50	0.47	6	105	0.001
Experiment 5					
Control isp-1(qm150);ctb-1(qm189);rwEx18[mec-7::rfp]					
Line 3	20.27	0.45	15	116	

isp-1(qm150);ctb-1(qm189);rwEx16[ceh-23+mec-7::rfp]					
Line 1	19.75	0.55	23	97	0.504
Line 3	22.48	0.53	5	111	0.001
Line 4	25.23	0.72	8	124	0.000
Experiment 6					
Control isp-1(qm150);ctb-1(qm189);rwEx18[mec-7::rfp]					
Line 3	22.04	0.37	7	117	
isp-1(qm150);ctb-1(qm189);rwEx16[ceh-23+mec-7::rfp]					
Line 1	20.40	0.44	15	106	0.016
Line 3	21.58	0.57	4	104	0.586
Line 4	26.31	0.61	9	107	0.000
ceh-23(ms23);isp-1(qm150) background					
Experiment 7					
					p-value (stratified log-rank test)
Wild-type	17.17	0.17	9	105	
rwIs21[ceh-23+mec-7::rfp]	17.34	0.22	28	105	0.488 (compared to wild-type)
ceh-23(ms23);isp-1(qm150);rwIs19[mec-7::rfp]	19.30	0.72	11	104	
ceh-23(ms23);isp-1(qm150);rwIs21[ceh-23+mec-7::rfp]	27.16	0.59	3	100	0.000 ((compared to *ceh-23(ms23);isp-1(qm150);rwIs19[mec-7::rfp]*
RNAi experiments					
					p-value (stratified log-rank test) compared to empty vector in corresponding background
Experiment 8					
Control rwEx18[mec-7::rfp]					
Line 3+empty vector	13.20	0.35	5	125	
Line 3+ ceh-23 RNAi	14.15	0.38	4	136	0.079
rwEx16[ceh-23+mec-7::rfp] (10ng/µl)					
Line 5+ empty vector	16.44	0.45	8	102	
Line 5+ ceh-23 RNAi	13.52	0.35	2	166	0.000
**Experiment 9**					
Control rwEx18[mec-7::rfp]					
Line 3+empty vector	13.63	0.36	5	128	
Line 3+ ceh-23 RNAi	12.80	0.28	6	162	0.065

rwEx16[ceh-23+mec-7::rfp] (10ng/µl)					
Line 5+ empty vector	15.51	0.44	4	111	
Line 5+ ceh-23 RNAi	13.03	0.31	2	143	0.000
**Experiment 10**					
Control rwEx18[mec-7::rfp]					
Line 3+empty vector	12.92	0.36	1	146	
Line 3+ ceh-23 RNAi	12.94	0.37	4	143	0.947
rwEx16[ceh-23+mec-7::rfp] (10ng/µl)					
Line 5+ empty vector	15.41	0.43	0	99	
Line 5+ ceh-23 RNAi	12.55	0.41	1	101	0.000
